# Cost of malaria treatment and health seeking behaviour of children under-five years in the Upper West Region of Ghana

**DOI:** 10.1371/journal.pone.0195533

**Published:** 2018-04-13

**Authors:** Maxwell Ayindenaba Dalaba, Paul Welaga, Abraham Oduro, Laata Latif Danchaka, Chieko Matsubara

**Affiliations:** 1 Navrongo Health Research Centre, Navrongo, Ghana; 2 Community Development Alliance Ghana, Wa, Ghana; 3 Bureau of International Medical Cooperation, National Centre for Global Health and Medicine, Tokyo, Japan; Centro de Pesquisas Rene Rachou, BRAZIL

## Abstract

**Background:**

There is limited knowledge on cost of treating malaria in children under-five years in northern Ghana which poses a challenge in determining whether interventions such as the National Health Insurance Scheme (NHIS) and Community-based Health Planning and Services (CHPS) have reduced the economic burden of malaria to households or not. This study examined the malaria care seeking and cost of treatment in children under-five years in the Upper West Region of Ghana.

**Methods:**

The study used a cross-sectional, quantitative design and data were collected between July and August 2016 in three districts in the Upper West Region of Ghana. A total of 574 women who had under-five children were interviewed. Socio-demographic characteristics of respondents, malaria seeking patterns for under-five children with malaria as well as direct medical and non-medical costs associated with treating under-five children with malaria were collected from the patient perspective. Analysis was performed using STATA 12.

**Results:**

Out of 574 women visited, about 63% (360) had children who had malaria and sought treatment. Most treatment was done at formal health facilities such as the health centres (37%) and the CHPS (35%) while 3% had self-treatment at home. The main reason for choice of place of treatment outside home was nearness to home (53%). The average direct medical and non-medical costs associated with treating an under-five child with malaria were US$4.13 and US$3.04 respectively. The average cost on transportation alone was US$2.64. Overall, the average direct medical and non-medical cost associated with treating an under-five child with malaria was US$4.91(range: minimum = US$0.13 –maximum = US$46.75). Children who were enrolled into the NHIS paid an average amount of US$4.76 compared with US$5.88 for those not enrolled, though the difference was not statistically significant (p-value = 0.15).

**Conclusions:**

The average cost to households in treating an under-five child with malaria was US$4.91. This amount is considerably high given the poverty level in the area. Children not insured paid a little over one US dollar for malaria treatment compared to those insured. Efforts to improve enrolment into the NHIS may be needed to reduce the cost of malaria treatment to households. Construction of more health facilities near to community members and at hard to reach areas will improve access to health care and reduce direct non-medical cost such as transportation costs.

## Introduction

Though there has been a global reduction in malaria burden, the disease remains a major public health problem particularly in sub-Saharan Africa (SSA) [1,2]. The number of malaria cases globally dropped from an estimated 262 million in 2000 to 214 million in 2015, representing an 18% decline [1,2]. In 2015, about 88% of the total global malaria cases and 90% of deaths occurred in SSA [1,2]. Malaria morbidity and mortality are particularly high in women and children less than five years old [1]. Globally, the number of malaria deaths in children aged under 5 years was estimated to be 306 000 in 2015 (range: minimum = 219 000– maximum = 421 000) and over 80% of the deaths occurred in SSA [1,2]. Malaria poses a significant burden to households and the economy of developing countries [3–6]. It has been estimated that the gross domestic product (GDP) of many developing countries could be reduced by 5–6% due to malaria [2,7]. In a study conducted by Secure et al [8] on the economic costs of malaria in children in three SSA countries, they reported costs per malaria episode to range from US$7.99 to US$ 229.24 in Ghana, from US$5.2 to US$137.74 in Tanzania, and from US$11.24 to US$287.81 in Kenya [8].

In Ghana, malaria is one of the leading causes of morbidity and mortality accounting for about 38% of outpatient visits and 27.3% of admissions in health facilities, 7.2% of all deaths on admission and 48.5% of under-five mortality in 2014 [2,9]. Malaria causes both direct and indirect burden to households which sometimes lead to catastrophic payment and poverty [4,10]. For instance, Novignon et al [11] reported that businesses in Ghana lost about US$6.58 million to malaria in 2014 and 90% of which were direct cost [11]. Also, in a study conducted in 2014 in the Kassena Nankana district of northern Ghana, the average cost incurred by households per malaria treatment was estimated at US$13.9 [12]. Similarly, a study conducted in 2016 by Tawiah et al [10] in the middle belt of Ghana reported average cost to households as US$14.61 per patient per fever/malaria episode [10].

The Government of Ghana has put in place strategies to improve access to healthcare and to reduce cost of healthcare. For instance, in 2003, the government of Ghana established the National Health Insurance Scheme (NHIS) to improve access to health care and reduce direct costs of treatment to households. The scheme accredits both public and private health facilities in the country to provide medical care at the point of service for those enrolled into the scheme [13]. The NHIS covers about 95% of diseases including malaria. Children under 18 years old whose parents or guardians are NHIS members, the aged (70 years and above) in the informal sector as well as indigents are exempted from paying premiums. All adults who do not contribute to the Social Security and National Insurance Trust (SSNIT) and who are mostly informal sector workers, have to pay an annual premium and processing fee in order to enrol into the NHIS. Annual premiums vary from district to district and the NHIS Act of 2012 (Act 852) stipulated that premium should range between a minimum of GH¢7.2 (US$4.8) and a maximum of GH¢48 (US$32.0). The premiums for workers who contribute to SSNIT and who are mostly formal sector workers are deducted directly from their SSNIT contributions (2.5% of SSNIT contribution) [13,14].

In addition, the government of Ghana introduced the free maternal care policy for pregnant women in 2008 through the national health insurance. With the free maternal policy, pregnant women are to be enrolled into the NHIS free of charge. They are not to pay registration and premium charges. The benefits includes free medical services and care provided to the newly born baby on the mother’s NHIS ticket for 90 days after which the baby must be registered with the NHIS [15].

To improve geographical access to health and to bridge equity gap, the government and other organizations such as Japan International Cooperation Agency (JICA) are expanding health care at the rural level through Community-based Health Planning and Services (CHPS). CHPS is a primary healthcare program where health workers are relocated into rural communities to provide primary healthcare services. The health workers operate from CHPS Centres (facilities) and also embark on outreach programmes by visiting community members at home to provide primary healthcare services [16–17].

There is however little evidence to show whether these interventions have improved access and reduced cost to seeking malaria care in under five children in Ghana. This study therefore examined access to malaria care among under-five children and how much households spent on malaria treatment in the Upper West Region of Ghana.

## Methods

### Study area

The study was conducted in the Upper West Region (UWR). Upper West Region is one of the 10 Regions in Ghana, located in the northern part of Ghana. Its population is 680,000 (2010), and it is one of the regions with a low population density in Ghana. The Region is bordered by Burkina Faso to its North and West. The major ethnic groups in the region are the Dagaati, Sissala, and Wala [18]. Subsistence agriculture is the mainstay of the people. The region is one of the poorest regions in Ghana with the highest malaria burden in the country [19]. The prevalence of fever among under-five children in the study area is 25% [19].

There is a total of 242 health facilities providing various types of services in the Upper West Region. There are three (3) district government hospitals, one (1) Regional hospital, two (2) Christian Health Association of Ghana (CHAG) hospitals and three (3) private hospitals. The rest are five (5) Polyclinics, sixty six (66) health centres, ten (10) clinics, one hundred and forty seven (147) CHPS Compounds and four (4) maternity homes [20].

### Study design

The study design was cross sectional and data were collected from July to August 2016. Quantitative methods were used in the data collection. We interviewed women of reproductive age who had under-five children.

This study was part of a bigger study that aimed at determining access to maternal and child health services, cost of providing primary health care services [17], as well as costs in seeking malaria care for under-five children in the Upper West Region of Ghana. Only households with under-five children who had malaria in the last one month prior to the survey and sought treatment were included in this study.

In cost of illness studies, data collection and analysis are carried out either from the societal perspective (including the patient and the health system/provider costs), health provider perspective (including costs incurred by the health provider) or patient perspective (the patient only or patient and care giver costs) [21,22]. This study adopted a patient perspective as used in similar studies [10,22–24] and estimated costs incurred by the household on under five child who had malaria as well as cost incurred by the care giver in seeking the care for the child such as transportation costs.

A structured questionnaire was used to collect data and respondents were asked questions on socio-demographic characteristics, household assets, health seeking and cost of treating the most recent under-five child who had malaria within the past 1 month and sought care/treatment.

### Sampling techniques

A multi stage method was used in selecting respondents for the study. At the first stage, we grouped all the 11 districts in the Upper West region by the three main ethnic groups. The Dagaati districts comprised of DBI, Jirapa, Lambussie, Lawra, Nadowli, Nandom; the Wala districts comprised of Wa East, Wa Municipality, Wa West; and the Sissala districts comprised of Sissala East and Sissala West.

In the second stage, we randomly selected one district from each of the ethnic groups. This method ensured a geographical representation of respondents across the region. Nadowli district was randomly selected to represent the Dagaati ethnic group, Wa West district was selected to represent the Wala ethnic group and Sissala East to represent Sissala ethnic group.

In the third stage, we randomly selected a sub-district within each district and then communities in the sub-district. Interviewers were then allocated to communities to identify respondents by moving from one household to another until they meet their sample target. A team of 5 interviewers were sent to each sampled community in a day. A target of 5–8 interviews was assigned to each interviewer depending on the size of the sampled community. The interviewers were assigned to different sections of the community. In each section of the community, households were then randomly selected by the interviewer going to the centre of the section and spinning a pen. The direction in which the pen pointed was followed and from which the first house was selected. Only one household with a child below five years was interviewed in a house. In case a house had more than one households with under-five children, one household was randomly selected. If there were more than two women with under-five children in a sampled household, only one of them was randomly selected for interview. The interviewer then moves to the nearest house/household. This was continued till the maximum number of respondents assigned to the interviewer was obtained for that community.

### Sample size

The sample size for this study was calculated by assuming that 50% of the women in the study districts had at least one child aged less than five years and with 95% confidence level [10,25], and design effect of 1.3 [26] to account for clustering effect in the districts would require a sample size of 500. Adjusting for a refusal rate of 15% yielded a sample size of 575.

### Data collection

Graduate level data collectors were recruited for the data collection. The selection criteria included ability to understand and speak the local language, familiarity with the environment and previous experience on data collection. Data collectors were trained on the study protocol, instruments and guidelines in conducting interviews. Data collectors interviewed women of reproductive age who had under-five children. The questions included socio-demographic characteristics of mothers/caregivers, household assets as well as health seeking and cost of treating the most recent under-five child who had malaria within the past 1 month and sought care/treatment (S1 Questionnaire). The episode of malaria was based on the report given by caregivers. Treatment could either be at the health facility or at home by buying drugs from the pharmacy or drug shops.

### Data processing and analysis

The data were double entered, cleaned and verified using EPI Data 6.1. Inconsistencies in entries between two data entry clerks who entered a questionnaire were checked from the source document and corrections made. Data were then transferred to STATA version 12 (STATA Corporation, College Station, Texas) for analysis. Additional data cleaning by way of identifying outliers, missing values and checking for consistency among variables were carried out by running frequencies and cross tabulations. The source documents were checked and in some cases phone calls were made to respondents to correct some inconsistencies. Basic frequencies, proportions and averages/means were estimated and presented in tables.

The data was analyzed as survey data, and we therefore used ‘svyset’ in STATA to identify the survey design characteristics with community as the primary sampling unit (clusters). We then prefixed the estimation commands with ‘svy:’ in STATA. For the purpose of this study, a total of 15 communities were randomly sampled from the three districts. Differences in mean cost of treating malaria were tested using Student’s t-test.

Direct and non-direct medical costs were calculated. The direct medical costs covered all out-of-pocket payments (OOP) for registration card, consultation, diagnosis, medicines and medical supplies on the patient; and direct non-medical costs included all out-of-pocket payments for transportation to and from health facilities (patient and caregiver) and other OOP or informal payments. Informal payments (“under the table payments”) are OOP payments made by patients/caregivers to the health staff for services and medical products that are officially free of charge at the health facility. The total direct and non-direct medical costs were summed and divided by the number of households that made expenditure to arrive at the average costs per treatment.

Catastrophic payments for malaria treatment were also calculated. Catastrophic payments occur when total OOP payments for health care exceeds a certain threshold of a household’s resources (income or expenditure) [4,27,28]. Thresholds for calculating catastrophic payments vary, usually ranging from 2.5% to 40% [4,27,28]. This study used a threshold of 5% as applied in other studies [4,29] and all households that spent more than 5% of their annual income to pay for the treatment of malaria were deemed to have made catastrophic payments. The annual income of households was calculated using estimated cost of yields or proceeds from agricultural products such as crops, poultry as well as from salaries or wages from business, income from investments or gifts (for the dataset used for the analysis, See S1 Data).

All costs were collected in Ghana Cedis (GH¢) and results presented in US$. The US$ conversion was based on the average exchange rate between January 2016- June 2016 (1US$ = 4GH¢).

### Ethical considerations

Ethical approval was obtained from the Navrongo Health Research Centre Institutional Review Board (Approval ID: NHRCIRB232) and the National Centre for Global Health and Medicine (NCGM), Japan (Approval ID: NCGM-G-0020510-00). Permission was also sought from the regional health directorate of UWR, district directorate of Wa West, Nadowli and Sissala East as well as chiefs of selected communities for the interviews. All the respondents were briefed on the study procedure and written informed consent was obtained. For the illiterate respondents, consenting was done in their preferred local language and those who agreed to participate thumb printed on the consent form.

## Results

### Background characteristics of survey respondents

Table 1 presents the background characteristics of respondents. A total of 574 women were interviewed. Out of the 574 respondents, about 76% (433) were between 20–34 years old, 61% (347) had never been to school and only 1% (3) had tertiary education. With regards to occupation, most of the women reported to be farmers (81%).

**Table 1:** Background characteristics of respondents interviewed.

Variable	Number (574)	Percent (%)
**Age group**		
15–19	23	4.04
20–34	433	75.96
35+	114	20.00
**Ethnicity**		
Wala	98	17.28
Sissala	201	35.45
Dagaati	242	42.68
Other	26	4.59
**Education**		
Never Been to School	347	60.88
Primary School	113	19.82
Junior High/Middle/Technical Sch	78	13.68
Secondary School/Senior High/O'level/A level	29	5.09
University/College/Polytechnic	3	0.53
**Occupation**		
Farmer	462	80.91
Industrial Worker	2	0.35
Home Industry/Artisan	52	9.11
Market Vender/Trader	26	4.55
NGO/Private Employee	2	0.35
Shop/Restaurant Owner or Worker	4	0.70
Civil Servant (Government Employment)	2	0.35
No Income Generation(Housekeeping)	17	2.98
Student	4	0.70
**NHIS status of respondents**		
Insured	484	84.76
Not insured	87	15.24
**Child with malaria**		
Yes	412	74.10
No	144	25.90
**NHIS status of child with malaria**		
Insured	350	85.00
Not insured	62	15.00

The study asked of any form of health insurance that the respondent had enrolled into during the period. In all, 85% (484) of respondents reported to have enrolled into the National Health Insurance Scheme (NHIS) at the time of interview and the rest were not enrolled into any form of health insurance. About 74% (412) reported that they had one child under five years old who had malaria in the past 1 month and sought care. Out of the 412 under five children who reported to have had malaria, 85% (350) of them paid the NHIS registration fee and were enrolled into the NHIS. The main reason for non- enrolment into the NHIS was basically due to lack of money given that registration fee per a child is about US$1.25.

### Malaria treatment seeking behaviour

Out of 412 children reported to have had malaria during the recall period, 87% (360) reported seeking care and provided information on where they sought the care. For the children who did not seek care, non-availability of money was given as the reason why care was not sought for them. The results showed that home treatment (self-treating at home using herbs and leftover drugs) for children with malaria illness was about 3% and most treatment was done at formal health facilities such as the hospitals, health centres, CHPS and private clinics. Most children got treatment for malaria illness at the health centres (37%) and the CHPS compounds (35%).

### Reasons for choosing place for malaria treatment

**Fig 1:**
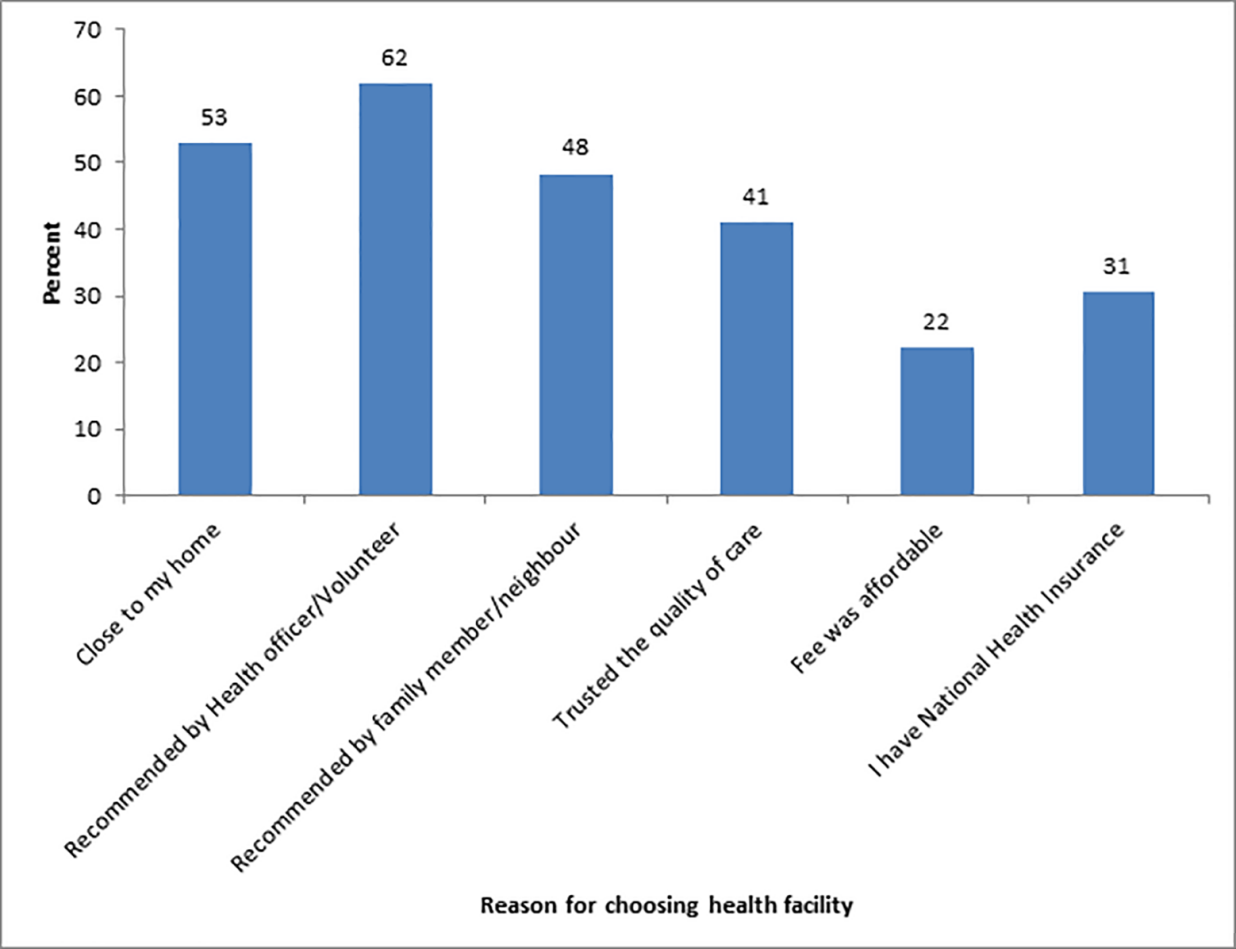
Percentage distribution of reasons for choosing place for malaria treatment.

When mothers were asked for reasons why they chose a particular health facility for treating the child’s last malaria, various reasons were provided for the choice of place for treatment. About 53% (233) mentioned nearness to home as the reason, 62% (276) were recommended by community health officer/volunteer, 48% (216) recommended by family members/neighbour, and trust in the quality of care was reported by 41% (183) as reason for a choice of place of treatment (Fig 1).

### Malaria treatment cost

Table 2 shows the malaria treatment cost. Out of the 360 children who had malaria and sought treatment 1% (255) incurred cost of treatment. Cost estimations were based on only patients who incurred cost for malaria treatment. The cost of treatment included direct medical costs such as out-of-pocket payments (OOP) for registration card, consultation, diagnosis, medicines and medical supplies; and direct non-medical costs such as OOP for transportation to and from health facilities and other OOP or informal payments.

**Table 2:** Malaria treatment cost (US$).

	Variable	Number that paid	Average cost (SD)	Minimum cost	Maximum cost
Medical cost	Registration card	54	1.32 (1.63)	0.25	7.50
	Consultation	7	1.79 (1.55)	0.50	5.00
	Diagnosis	31	2.09 (2.44)	0.25	12.50
	Drugs	132	4.03 (5.17)	0.25	37.50
	Medical supplies	10	2.93 (2.01)	0.25	5.50
***Sub-Total***	*** ***	172	4.13 (5.55)	0.25	45.50
Non-medical cost	Transportation	168	2.64 (2.70)	0.25	15.00
	Other non-medical payments	28	3.50 (4.66)	0.13	25.00
***Sub-Total***	*** ***	178	3.04 (3.46)	0.13	25.00
**Overall (medical and non-medical costs)**		255	4.91 (5.81)	0.13	46.75

SD = Standard deviation

Of those who incurred cost of treating malaria, about 67% (172) incurred direct medical cost and 70% (178) incurred direct non-medical cost. The average amount paid on direct medical cost was US$4.13 whiles the direct non-medical cost was US$3.04. Most of the direct medical cost was on drugs such as antimalarial and paracetamol and 132 respondents paid for these drugs at an average cost of US$4.03. About 66% (168) incurred cost on transportation to and from the health facilities and the average expenditure was US$2.64. The average amount spent on other non-medical payments/informal payments was US$3.50. Some patients incurred both direct medical costs and non-medical cost, others incurred only direct medical costs but no expenditure on non-medical cost and vice versa for some patients.

Overall, the average direct medical and direct non-medical cost associated with the treatment of a child’s malaria by households was estimated at US$4.91(range: minimum = US$0.13 –maximum = US$46.75). As shown in Table 3, the children who were enrolled into the NHIS paid less (US$4.76) compared to those not insured (US$5.88), though the difference is not statistically significant (p value = 0.1519).

**Table 3:** Malaria treatment cost (US$) by insurance status.

	Variable	Number that paid	Average cost (SD)	Minimum cost	Maximum cost
**Insurance status of child**	Insured	222	4.76 (5.41)	0.13	46.90
	Not insured	33	5.88 (8.00)	0.50	38.25
	Total	255	4.91 (5.81)	0.13	46.90

SD = Standard deviation

### Catastrophic payments from malaria treatment

Households that spent more than 5% of their annual income to pay for the treatment of malaria were deemed to have catastrophic payment as a result of treating malaria. About 5% (12) households from a total of 255 households who incurred cost for malaria treatment spent more than 5% of their annual household income to treat malaria and were deemed to have catastrophic payment as a result of malaria treatment.

## Discussion

The main aim of the study was to determine access to under-five malaria care and how much households spent on malaria treatment in the Upper West Region of Ghana.

The results of the study showed most children received malaria treatment from formal health providers. This finding indicates some improvement in seeking malaria treatment as other previous studies in Ghana reported that majority of respondents sought malaria care from drug stores [10,24]. Improvement in treating children with malaria at the formal health facilities could be partly attributed to the NHIS as most of the children (72%) were enrolled into the NHIS. The positive effect of health insurance status on seeking care at formal health care facilities has been reported in other studies [5,30–33]. In addition, a possible reason for high usage of formal health facilities could be reduction in distance to seek care due to government efforts to increase the availability of health facilities close to community members such as the CHPS. For instance, 53% of respondents in our study cited nearness to home as a reason for choice of health facility for care.

Overall, the average cost of treating an under-five child with an episode of malaria was US$4.91. This amount is higher than the direct cost of malaria treatment estimated in similar studies conducted in other parts of Ghana [10,12]. For instance, Tewiah et al estimated mean direct cost per fever case to be US$2.76 [10]. The cost of treatment of malaria in our study is relatively high considering the poverty level of the study area. According to the Ghana Living Standard Survey report (GLSS6), Upper West Region has the highest proportion of households in the lowest quintile (55.7%) compared to the other regions [34]. As it has been revealed in our study, about 5% households spent 5% of their annual income in treating an episode of malaria which is considered catastrophic payment. In addition, malaria is endemic in the study area with a prevalence of 37.8% in 2015 (one of the highest prevalence of malaria in Ghana) [35]. Multiple malaria episodes for the same child or in the same household within a year is not uncommon [35,36] and this situation can impose greater burden to the households.

The study findings showed that although OOP payments were made on both the insured and uninsured children, expenditures were higher among the uninsured (US$5.88) than the insured children (US$4.76). Though the cost difference was not statistically significant between the insured and the uninsured, given the poverty level in the study area, any small difference in expenditure (a difference of US$1.12 between the insured and uninsured) is very relevant to the household and can change health seeking behaviour and burden to households. Also, there were very few patients who were not enrolled, so a larger sample size might be required to determine any significant difference between unenrolled and enrolled children. Therefore, the effect of NHIS enrolment in reducing the cost of treatment cannot be underestimated. For instance, a study in Ghana showed that, prior to the introduction of the NHIS, OOP healthcare payments had impoverishing effect on Ghanaians with a higher proportion of the population pushed into poverty due to OOP healthcare payments [37]. In addition, prior to the introduction of the NHIS, the cost of malaria treatment in northern Ghana was reported as US$6.39 [24]. Our study findings of less expenditure on the insured compared to the uninsured is consistent with findings of some studies conducted in Ghana where the uninsured paid more for malaria care than the insured [14,38,39]. However, enrolment of households into the NHIS is not encouraging given that as at 2016, only 40% of Ghanaians were enrolled into the scheme with valid membership cards [40]. It has been reported that the main reason for low enrolment into the NHIS is affordability of the premium and poor households are less covered [30,41–45].Therefore, innovative methods are required to improve enrolment into the NHIS, especially the poor and vulnerable.

Despite efforts by the government of Ghana and JICA to expand health facilities such as CHPS compounds in the study area, the average transportation cost to and from health facilities was still high (US$2.64). A possible reason could be that the health facilities are not evenly distributed making accessibility difficult and expensive to reach. Transportation cost has been reported in other studies as a significant cost of seeking malaria treatment and influences health seeking [5,6,12,46]. Attention is therefore needed to reduce transportation costs in order to improve access to health care.

In addition to transportation as a direct non-medical cost, about 10% (28) of the respondents who incurred cost in treating malaria indicated that they made informal payments at an average cost of US$3.50. Some of the informal payments could be made as appreciation to the health staff for good services, and sometimes patients or caregivers can also be induced or coerced to make such informal payments. However, we did not ask for the reasons for the informal payments in this study.

### Study limitations

One possible limitation of the study is recall bias. Given that costs were assessed based on verbal reporting of costs by the respondents without prove of receipts, there could be a possibility of over estimation or under estimation of costs. However, because the recall period was short (1 month), and the probing techniques used, we do not expect much recall issues to affect our results.

The history of malaria was based on the report given by caregivers on malaria and could be possible that some were really not malaria cases. However, given that malaria is endemic in the study area, we assumed that knowledge about malaria signs and symptoms by caregivers was high and therefore a high probability of giving accurate information. The use of caregiver recollection of fever or malaria in assessing malaria treatment and cost has been used in many studies [5,24,25,47,48].

Also, the study did not assess malaria by severity since the emphases was just on cost of malaria treatment. However, we do acknowledge this as a limitation given that there could be cost variations and treatment seeking between moderate and severe malaria.

The study did not include indirect costs (productivity lost due to illness) and intangible costs (quality of life due to illness) in the analysis which we acknowledge as a limitation to the study and therefore an underestimation of the cost of treatment. Though intangible cost is an important cost component, it is usually complex and difficult to measure especially in settings were illiteracy level is high and therefore this cost component is not usually included in most costing studies.

## Conclusions

The average cost among households who incurred any cost in treating children with malaria was US$4.91. This amount is considerably high given the relatively high poverty level in the Upper West Region of Ghana. Children not insured paid a little over one US dollar for malaria treatment compared to those insured. Treatment cost can pose substantial burden to households, particularly poor households and can adversely affect health seeking and lead to catastrophic payment and poverty.

Efforts to improve enrolment into the NHIS may be needed to reduce the cost of malaria treatment to households and access to formal health care. The average transportation cost to and from health facilities was still high (US$2.64) and therefore needs attention. The construction of more health facilities at hard to reach areas will improve access to health care and reduce direct non-medical cost including transportation costs.

## Supporting information

S1 Questionnaire The questionnaire used for the study. (DOC)

S1 Data The data used in the study analysis. (DTA)

## Abbreviations

CHAG: Christian Health Association of Ghana
CHPS: Community-based Health Planning and Services
DBI: Daffiama Bussie Issa
GDP: Gross Domestic Product
GLSS: Ghana Living Standard Survey
NHIS: National Health Insurance Scheme
OOP: Out-of-pocket Payment
SSNIT: Social Security and National Insurance Trust
SSS: Sub-Saharan Africa
UWR: Upper West Region
