# Supplementary material for: Cost of malaria treatment and health seeking behaviour of children under-five years in the Upper West Region of Ghana
Source: PLoS One. 2018 Apr 13;13(4):e0195533. doi: 10.1371/journal.pone.0195533 (PMC5898715; doi:10.1371/journal.pone.0195533)
Supplement: S1 Questionnaire — (DOC) [file pone.0195533.s001.doc]

**ID number:**

**Research on health financing schemes and trend of the consultation and payment of the patient aiming universal health coverage (UHC) in Africa (UHC study)**

**Ghana Questionnaire (Ver.1.1)** 20 May 2016

Date of interview; ______, ________, 2016

Location of interview: ___________ district, ___________ CHPS, _________community

Name of interviewee/Respondent: ___________________________

Contact number of interviewee____________________

Name of interviewer_________________________________________

(There is a separate explanations/informed consent sheet)

1. First, I will ask about your inclusion criteria.

1-1. Deliver in the last year (2015)?

1) Yes ➭ Please go to 1-2.

2) No ➭ Your household does not fall within the inclusion criteria of this study. Thank you very much!

1-2. Type of delivery

1) Spontaneous (normal) delivery 2) Caesarian delivery

3) Referred to the hospital (Specify the reason_____________________)

1-3. Do you have a child/ children under 5-years-old who suffered diseases associated with fever including malaria in the last year (2015)?

1) Yes 2) No

1-4. If yes, What is the name of the child? [Child 1] _____________________

Age _______years, Date of birth: (DD)______, (MM)_______, (YY)__________

What is the name of the child? [Child 2] _____________________

Age _______years, Date of birth: (DD)______, (MM)_______, (YY)__________

1. Second, I will ask about your basic information.

2-1. Age: ______ years, Date of birth: (DD)______, (MM)_______, (YY)__________

2-2. Ethnic group

1. Wala 2) Sissala 3) Dagaaba 4) Others (Specify_____________________)

2-3. Your highest level of formal education received

1) Never been to school 2) Primary school 3) Junior high/Middle /technical school

4) Secondary school school/senior high/O’Level,/ A’ level 5) University/college/Polytechnic

6) Others (Specify_____________________)

2-4. Your partner’s highest level of formal education received

1) Never been to school 2) Primary school 3) Junior high/Middle/ technical school

4) Secondary school /senior high/O’Level,/ A’ level 5) University/college/Polytechnic

6) Others (Specify_____________________)

2-5. Your occupation

1. Farmer 2) Fisherman 3) Industrial worker 4) Home industry/Artisan 5) Market vender/Trader

6) NGO/Private employee 7) Shop/Restaurant owner or worker

8) Civil servant (Government employee) 9) No income generation (e.g. Housekeeping) 11) student 12) Others (Specify__________________)

2-6. Your partner’s occupation

1. Farmer 2) Fisherman 3) Industrial worker 4) Home industry/Artisan 5) Market vender/Trader

6) NGO/Private employee 7) Shop/Restaurant owner or worker

8) Civil servant (Government employee) 9) No income generation (e.g. Housekeeping) 11) student 12) Others (Specify__________________)

2-7. Total number of your household members: _____persons

2-8. Residential status: 1) Permanent resident 2) Migrant

2-9. Annual income of your household (including the income of your family members):

Agriculture

| **Types of crops** | **How many times of harvest per year?** | **How many bags did you harvest in one time?** | **How many bags did you sell in one time?** | **Types of bags?** | **How many bags of fertilizer did you use?** | **How many bags of herbicide did you use?** |
| --- | --- | --- | --- | --- | --- | --- |
| **2-9-1**  **Rice** | 2-9-1-1  times | 2-9-1-2  bags | 2-9-1-3  bags | 2-9-1-4 | 2-9-1-5  bags | 2-9-1-6  bags |
| **2-9-2**  **Millet** | 2-9-2-1  times | 2-9-2-2  bags | 2-9-2-3  bags | 2-9-2-4 | 2-9-2-5  bags | 2-9-2-6  bags |
| **2-9-3**  **Sorghum** | 2-9-3-1  times | 2-9-3-2  bags | 2-9-3-3  bags | 2-9-3-4 | 2-9-3-5  bags | 2-9-3-6  bags |
| **2-9-4**  **Shea butter** | 2-9-4-1  times | 2-9-4-2  bags | 2-9-4-3  bags | 2-9-4-4 | 2-9-4-5  bags | 2-9-4-6  bags |
| **2-9-5**  **Groundnut** | 2-9-5-1  imes | 2-9-5-2  bags | 2-9-5-3  bags | 2-9-5-4 | 2-9-5-5  bags | 2-9-5-6  bags |
| **2-9-6**  **Cashew nut** | 2-9-6-1  times | 2-9-6-2  bags | 2-9-6-3  bags | 2-9-6-4 | 2-9-6-5  bags | 2-9-6-6  bags |
| **2-9-7**  **Other____________** | 2-9-7-1  times | 2-9-7-2  bags | 2-9-7-3  bags | 2-9-7-4 | 2-9-7-5  bags | 2-9-7-6  bags |
| **2-9-8**  **Other____________** | 2-9-8-1  times | 2-9-8-2  bags | 2-9-8-3  bags | 2-9-8-4 | 2-9-8-5  bags | 2-9-8-6  bags |
| **2-9-9**  **Other___________** | 2-9-9-1  times | 2-9-9-2  bags | 2-9-9-3  bags | 2-9-9-4 | 2-9-9-5  bags | 2-9-9-6  bags |

Fishery:

2-10-1. What is your point of participation in fish business? 1) Fish catch 2) Retailing/market

2-10-2. How many bucket of fish did you obtain per week? ____________times

2-10-3. In average, how much was one bucket of fish? _____________ Gh¢

2-10-4. How many months did you catch/sale fish per year? ____________months

Poultry:

| **Types of poultry** | **How many do you own?** | **How many did you sell last year?** | **How much was one on average?** | **How many of bags did you feed per year?** | **Types of bags** |
| --- | --- | --- | --- | --- | --- |
| **2-11-1**  **Cattle/Milk cows or bulls?** | 2-11-1-1 | 2-11-1-2 | 2-11-1-3 | 2-11-1-4  bags | 2-11-1-5 |
| **2-11-2**  **Horses, donkeys or mules?** | 2-11-2-1 | 2-11-2-2 | 2-11-2-3 | 2-11-2-4  bags | 2-11-2-5 |
| **2-11-3**  **Goats?** | 2-11-3-1 | 2-11-3-2 | 2-11-3-3 | 2-11-3-4  bags | 2-11-3-5 |
| **2-11-4**  **Pigs?** | 2-11-4-1 | 2-11-4-2 | 2-11-4-3 | 2-11-4-4  bags | 2-11-4-5 |
| **2-11-5**  **Rabbits?** | 2-11-5-1 | 2-11-5-2 | 2-11-5-3 | 2-11-5-4  bags | 2-11-5-5 |
| **2-11-6**  **Grasscutter?** | 2-11-6-1 | 2-11-6-2 | 2-11-6-3 | 2-11-6-4  bags | 2-11-6-5 |
| **2-11-8**  **Sheep?** | 2-11-7-1 | 2-11-7-2 | 2-11-7-3 | 2-11-7-4  bags | 2-11-7-5 |
| **2-11-9**  **Chiekens?** | 2-11-8-1 | 2-11-8-2 | 2-11-8-3 | 2-11-8-4  bags | 2-11-8-5 |
| **2-11-10**  **Guinea fouls?** | 2-11-9-1 | 2-11-9-2 | 2-11-9-3 | 2-11-9-4  bags | 2-11-9-5 |
| **2-11-11**  **Other1________** | 2-11-10-1 | 2-11-10-2 | 2-11-10-3 | 2-11-10-4  bags | 2-11-10-5 |
| **2-11-12**  **Other2________** | 2-11-11-1 | 2-11-11-2 | 2-11-11-3 | 2-11-11-4  bags | 2-11-11-5 |

Others:

|  | **How much did you earn per months?** | **How many months did you work for the job?** |
| --- | --- | --- |
| **2-12-1 Salary/wages for business not owned by your family (including gov. jobs)** | Gh¢ | months |
| **2-12-2 Income from business you or your family own (e.g. shops, restaurants)** | Gh¢ | months |
| **2-12-3 Income from renting house (landloard income)** | Gh¢ | months |
| **2-12-4 Money sent by someone (e.g. your relative either abroad or in Ghana)** | Gh¢ | months |
| **2-12-5 Income from investiments such as interest from banking, payment from bonds, etc.** | Gh¢ | months |
| **2-12-6 Pension, social security benefits or any other transfer:** | Gh¢ | months |
| **2-12-7 Other1_______________________________** | Gh¢ | months |
| **2-12-7**  **Other 2______________________________** | Gh¢ | months |

3. Thirdly, we will ask about you and your child’s (____________’s) health insurance status at the time of the febrile disease.

In case more than two children suffered last year, ask the most recent case. Name of the child _________

| Are **you** a member of the | 3-1-1  SSNIT? (Social Security and National Insurance Trust)?  1-Yes 2-No | 3-2-1  NHIS? (National Insurance Health Scheme)  1-Yes 2-No | 3-3-1  Private (voluntary) health insurance?  1-Yes 2-No | 3-4-1  Any other scheme of insurance?  1-Yes 2-No |
| --- | --- | --- | --- | --- |
| If yes,  When did you participate?  Check the insurance card | 3-1-2  DD/MM/YYYY | 3-2-2  DD/MM/YYYY | 3-3-2  DD/MM/YYYY | 3-4-2  DD/MM/YYYY |
| Did you benefit from the health insurance during your pregnancy and delivery? | 3-1-3  1-Yes 2-No | 3-2-3  1-Yes 2-No | 3-3-3  1-Yes 2-No | 3-4-3  1-Yes 2-No |

| Is **your child** a member of the | 3-5-1  SSNIT? (Social Security and National Insurance Trust)? 1-Yes 2-No | 3-6-1  NHIS? (National Insurance Health Scheme)  1-Yes 2-No | 3-7-1  Private (voluntary) health insurance?  1-Yes 2-No | 3-8-1  Any other scheme of insurance?  1-Yes 2-No |
| --- | --- | --- | --- | --- |
| If yes,  When did your child participate?  Check the insurance card | 3-5-2  DD/MM/YYYY  _____________ | 3-6-2  DD/MM/YYYY  ____________ | 3-7-2  DD/MM/YYYY  _____________ | 3-8-2  DD/MM/YYYY  _____________ |
| Did your child benefit from the health insurance during febrile disease? | 3-5-3  1-Yes 2-No | 3-6-3  1-Yes 2-No | 3-7-3  1-Yes 2-No | 3-8-3  1-Yes 2-No |

3-6. If you and/or your child has never been registered health insurance, why?

| **3-6-1 Premium is too high** | **1-Yes 2-No** |
| --- | --- |
| **3-6-2 Do not have confidence in operators of the schemes** | 1-Yes 2-No |
| **3-6-3 Covered by other alternatives** | 1-Yes 2-No |
| **3-6-4 No knowledge of any scheme** | 1-Yes 2-No |
| **3-6-5 Do not know where to register** | 1-Yes 2-No |
| **3-6-6 Registration office is too far** | 1-Yes 2-No |
| **3-6-7 Do not need health insurance** | 1-Yes 2-No |
| **3-6-8 Health insurance does not cover the services I need** | 1-Yes 2-No |
| **3-6-9 Health insurance does not cover the facilities I use** | 1-Yes 2-No |
| **3-6-10 No money** | 1-Yes 2-No |
| **3-6-11 Others** | Specify__________________________ |

4. Fourth, we will ask about your last child delivery in the last year (2015).

4-1. Date of the **last delivery** : (Date)________, (Month)__________, (Year)__________

4-2. Where did you deliver the last child?

1. Home (No visit) 2) CHPS compound 3) Health centre 4) Polyclinic

5) District hosp. 6) Regional hosp. 7) Teaching hosp. 8) Mission Hosp/Clinic/private clinic

9) Others (Specify______________)

4-3. Why did you choose to deliver in above location? (Answer to all 8 questions below.)

| **4-3-1 Because it is close from my home** | **1-Yes 2-No** |
| --- | --- |
| **4-3-2 Because I was recommended by community health volunteer(s)** | 1-Yes 2-No |
| **4-3-3 Because I was recommended by community health officer(s)** | 1-Yes 2-No |
| **4-3-4 Because I was recommended by my family** | 1-Yes 2-No |
| **4-3-5 Because I was recommended by my neighbor(s)** | 1-Yes 2-No |
| **4-3-6 Because I trusted the quality of care** | 1-Yes 2-No |
| **4-3-7 Because the fee was affordable** | 1-Yes 2-No |
| **4-3-8 Because I have national health insurance** | 1-Yes 2-No |
| **4-3-9 Other reasons: Please specify:_______________________________________** | |

4-4. How much did you pay for

| 4-4-1  Registration card/ folder at the HF? | 4-4-2  Consultation? | 4-4-3  Diagnosis (x-ray, lab, etc.)? | 4-4-4  Drugs? | 4-4-5  Medical supplies? | 4-4-6  Overall treatment or services received? | 4-4-7  Informal payment? |
| --- | --- | --- | --- | --- | --- | --- |
| Gh¢ | Gh¢ | Gh¢ | Gh¢ | Gh¢ | Gh¢ | Gh¢ |

4-4-8. Any other payment apart from what is stated before? _____________________GHC

| 4-5-1.  How much did you pay to travel there and return? | 4-5-2.  How much time did you take to travel to and from the facility? | 4-5-3.  How much time did you spend at the health facility? (excluding admission) | |
| --- | --- | --- | --- |
| Gh¢ | min | hr(s) | min |

| 4-6-1.  Where you admitted to the HF? | 4-762.  If yes, how much did you pay for staying in the HF? |
| --- | --- |
| 1- Yes 2- No | Gh¢ |

4-7. Who pays for the largest portions of your last delivery expenses including consultations and hospital stays (if any)?

1) Household member (Specify______________) 2) Other relative 3) Government 4) Employer 5) Household member’s employer 6) Health insurance 7) Other (Specify______________)

4-8. Did you receive ANC?

1) Yes 2) No

4-9. If yes, how many times did you receive ANC? ______________time(s)

4-10. Where did you receive ANC? (Answer all 7 questions below, please)

| **1) Home (Home visit by CHO)** | **1 Yes 2 No** | **times** |
| --- | --- | --- |
| **2) CHPS compound** | 1 Yes 2 No | times |
| **3) Health centre** | 1 Yes 2 No | times |
| **4) Polyclinic** | 1 Yes 2 No | times |
| **4) District hospital** | 1 Yes 2 No | times |
| **5) Regional hospital** | 1 Yes 2 No | times |
| **6) Teaching hospital** | 1 Yes 2 No | times |
| **7)Private clinic** | 1 Yes 2 No | times |
| **7) Others (Specify________________)** | 1 Yes 2 No | times |

4-11. How much did you pay at the time of ANC **in place1_______________** for

| 4-11-1  Registration card/ folder at the HF? | 4-11-2  Consultation? | 4-11-3  Diagnosis (x-ray, lab, etc.)? | 4-11-4  Drugs? | 4-11-5  Medical supplies? | 4-11-6  Overall treatment or services received? | 4-11-7  Informal payment? | 4-11-8  Transportation to travel there and return? |
| --- | --- | --- | --- | --- | --- | --- | --- |
| Gh¢ | Gh¢ | Gh¢ | Gh¢ | Gh¢ | Gh¢ | Gh¢ | Gh¢ |

4-11-9. Any other payment apart from what is stated before? _____________________ Gh¢

4-12. How much did you pay at the time of ANC **in place2_______________** for

| 4-12-1  Registration card/ folder at the HF? | 4-12-2  Consultation? | 4-12-3  Diagnosis (x-ray, lab, etc.)? | 4-12-4  Drugs? | 4-12-5  Medical supplies? | 4-12-6  Overall treatment or services received? | 4-12-7  Informal payment? | 4-12-8  Transportation to travel there and return? |
| --- | --- | --- | --- | --- | --- | --- | --- |
| Gh¢ | Gh¢ | Gh¢ | Gh¢ | Gh¢ | Gh¢ | Gh¢ | Gh¢ |

4-12-9. Any other payment apart from what is stated before? _____________________ Gh¢

4-13. Did you receive PNC? 1) Yes 2) No

4-13-1. If yes, did you receive PNC within 7 days? 1) Yes 2) No

4-13-2 within 30 days 1) Yes 2) No

4-14. How much did you pay at the time of PNC **in place1_______________** for

| 4-14-1  Registration card/ folder at the HF? | 4-14-2  Consultation? | 4-14-3  Diagnosis (x-ray, lab, etc.)? | 4-14-4  Drugs? | 4-14-5  Medical supplies? | 4-14-6  Overall treatment or services received? | 4-14-7  Informal payment? | 4-14-8  Transportation to travel there and return? |
| --- | --- | --- | --- | --- | --- | --- | --- |
| Gh¢ | Gh¢ | Gh¢ | Gh¢ | Gh¢ | Gh¢ | Gh¢ | Gh¢ |

4-14-9. Any other payment apart from what is stated before? _____________________ Gh¢

4-15. How much did you pay at the time of PNC **in place2________________** for

| 4-15-1  Registration card/ folder at the HF? | 4-15-2  Consultation? | 4-15-3  Diagnosis (x-ray, lab, etc.)? | 4-15-4  Drugs? | 4-15-5  Medical supplies? | 4-15-6  Overall treatment or services received? | 4-15-7  Informal payment? | 4-15-8  Transportation to travel there and return? |
| --- | --- | --- | --- | --- | --- | --- | --- |
| Gh¢ | Gh¢ | Gh¢ | Gh¢ | Gh¢ | Gh¢ | Gh¢ | Gh¢ |

4-15-9. Any other payment apart from what is stated before? _____________________ Gh¢

**5. Fifth, we will ask about your child(ren) under 5-years-old who suffered diseases associated with fever including malaria in the last year (2015).**

In case more than 2 children suffered, ask the most recent case name of the child _______________

5-1. Date of _____________’s febrile diseases: (Date) _____, (Month)_______, (Year)__________

5-2. Where did your child ______________ get treatment for the febrile disease?

1. Home (No visit) 2) CHPS compound 3) Health centre 4) Polyclinic

5) District hosp. 6) Regional hosp. 7) Teaching hosp. 8) Mission hosp./ clinic

9) Pharmacy/chemical shop 10) Traditional healer’s home 11) private clinic 12) Others (Specify______________)

5-3. Why did you choose to get treatment in above location? (Answer to all 8 questions.)

| **5**-3-1 Because it is close from my home | 1-Yes 2-No |
| --- | --- |
| **5**-3-2 Because I was recommended by community health volunteer(s) | 1-Yes 2-No |
| **5**-3-3 Because I was recommended by community health officer(s) | 1-Yes 2-No |
| **5**-3-4 Because I was recommended by my family | 1-Yes 2-No |
| **5**-3-5 Because I was recommended by my neighbor(s) | 1-Yes 2-No |
| **5**-3-6 Because I trusted the quality of care | 1-Yes 2-No |
| **5**-3-7 Because the fee was affordable | 1-Yes 2-No |
| **5**-3-8 Because I have national health insurance | 1-Yes 2-No |
| **5**-3-9 Other reasons: Please specify:_______________________________________ | |

5-4. How much did you pay for

| 5-4-1  Registration card/ folder at the HF? | 5-4-2  Consultation? | 5-4-3  Diagnosis (x-ray, lab, etc.)? | 5-4-4  Drugs? | 5-4-5  Medical supplies? | 5-4-6  Overall treatment or services received? | 5-4-7  Informal payment? |
| --- | --- | --- | --- | --- | --- | --- |
| Gh¢ | Gh¢ | Gh¢ | Gh¢ | Gh¢ | Gh¢ | Gh¢ |

5-4-8. Any other payment apart from what is stated before? _____________________Gh¢

| 5-5-1.  How much did you pay to travel there and return? | 5-5-2.  How much time did you take to travel to and from the facility? | 5-5-3.  How much time did you spend at the health facility? (excluding admission) | |
| --- | --- | --- | --- |
| Gh¢ | min | hr(s) | min |

| 5-8-1.  Did your child admitted to the HF? | 5-8-2.  If yes, how much did you pay for staying in the HF? |
| --- | --- |
| 1- Yes 2- No | Gh¢ |

5-9. Who paid for the largest portion of your last febrile disease expenses including consultations and hospital stays (if any)?

1) Household member (Specify______________) 2) Other relative 3) Government 4) Employer 5) Household member’s employer 6) Health insurance 7) Other (Specify______________)

6. Sixth, we will ask about your satisfaction and comments on the quality of service given during the last ***delivery*** in the past 1 year.

6-1. How would you rate the quality of service you have received?

| 4- Excellent | 3- Good | 2- Fair | 1- Poor |
| --- | --- | --- | --- |

6-2. Did you get the kind of service you wanted?

| 1- No, definitely | 2- No, not really | 3- Yes, generally | 4- Yes, definitely |
| --- | --- | --- | --- |

6-3. To what extent has the service given during the delivery met your needs?

| 4- Almost all of my needs have been met | 3- Most of my needs have been met | 2- Only a few of my needs have been met | 1- None of my needs have been met |
| --- | --- | --- | --- |

6-4. If a friend were in need of similar help, would you recommend our program to her?

| 1- No, definitely not | 2- No, I don’t think so | 3- Yes, I think so | 4- Yes, definitely |
| --- | --- | --- | --- |

6-5. How satisfied are you with the amount of help you have received?

| 1- Quite dissatisfied | 2- Indifferent or mildly dissatisfied | 3- Mostly satisfied | 4- Very satisfied |
| --- | --- | --- | --- |

6-6. Have the services you received helped you to deal more effectively with your problems?

| 4- Yes, they helped a great deal | 3- Yes, they helped | 2- No, they really didn’t help | 1- No, they seemed to make things worse |
| --- | --- | --- | --- |

6-7. In an overall, general sense, how satisfied are you with the service you have received?

| 4- Very satisfied | 3- Mostly satisfied | 2- Indifferent or mildly dissatisfied | 1- Quite dissatisfied |
| --- | --- | --- | --- |

6-8. If you were to seek help again, would you come back to our program?

| 1- No, definitely not | 2- No, I don’t think so | 3- Yes, I think so | 4- Yes, definitely |
| --- | --- | --- | --- |

6-9. If you have any comments, please specify________ ___

7. Lastly, seventh, we will ask about your household status

7-1. What is the main source of drinking water for members of your household?

PIPED WATER: 11) Piped into dwelling 12) Piped to yard/ plot 13) Public tap/ standpipe

21) TUBE WELL OR BOREHOLE

DUG WELL: 31) Protected well 32) Unprotected well

WATER FROM SPRING: 41) Protected spring 42) Unprotected spring

51) RAINWATER

61) TANKER TRUCK

71) CART WITH SMALL TANK(motor king)

81) SURFACE WATER (RIVER/LAKE/POND/STREAM/CANAL/IRRIGATION CHANNEL)

91) BOTTLED WATER

92) SACHET WATER

96) OTHER (Specify )

7-2. What is the main source of water used by your household for other purposes such as cooking?

PIPED WATER: 11) Piped into dwelling 12) Piped to yard/plot 13) Piped to neighbor

14) Public tap/ Standpipe

21) Tube well or Borehole

Dug WELL: 31) Protected well 32) Unprotected well

WATER FROM SPRING: 41) Protected spring 42) Unprotected spring

51) RAINWATER

61) TANKER TRUCK

71) CART WITH SMALL TANK

81) SURFACE WATER (RIVER/ LAKE/POND/STREAM/CANAL/IRRIGATION CHANNEL)

91) OTHER (Specify )

7-3. Where is that water source located?

1) In own dwelling

2) In own yard/ plot

3) Elsewhere

7-4. What kind of toilet facility do members of your household usually use?

If not possible to determine, ask permission to observe the facility.

FLUSH OR POUR FLUSH TOILET: 11) Flush to piped sewer system 12) Flush to septic tank

13) Flush to pit latrine 14) Flush to somewhere else 15) Flush don’t know where

PIT LATRINE: 21) Ventilated improved pit latrine 22) Pit latrine with slab

23) Pit latrine without slab/ open pit

41) BUCKET TOILET

51) HANGING TOILET/ HANGING LATRINE

61) NO FACILITY/ BUSH/ FIELD

71) OTHER (Specify )

7-5. What type of fuel does your household ***mainly*** use for cooking (Select the best one)?

1) Electricity 2) LPG 3) Natural gas 4) Biogas 5) Kerosene 6) Coal, Lignite 7) Charcoal 8) Wood

9) Straw/ Shrubs/ Grass 10) Agricultural corp 11) Animal dung 95) No food cooked in household

96) OTHER (Specify )

7-6. Do you share this toilet facility with other households?

1) Yes, other households only 2) Yes, public 3) no

7-7.What is the main material of the floor of your household?

NATURAL FLOOR: 11) Earth/Sand/Clay 12) DUNG

RUDIMENTARY FLOOR: 21) Wood planks

FINISHED FLOOR: 31) Paquet or Polished Wood 32) Vinyl or Asphalt strips

33) Ceramic / Marble / Porcelain tiles / Terrao 34) Cement

35) Woolen carpet / Synthetic carpet 36) Linoleum / Rubber carpet

96) OTHER (Specify ______________ )

7-8. What is the main material of the roof of your household?

NATURAL ROOFING 11) No roof 12) Thatch

RUDIMENTARY ROOFING 21) Rustic mat 22) Palm / Bamboo 23) Wood planks 24) Cardboard

FINISHED ROOFING 31) Metal 32) Wood 33) Calamine / Cement fiber 34) Ceramic / Brick tiles 35) Cement 36) Roofing shingles 37) Asbestos / Slate roofing sheets

96) OTHER (Specify ___________ )

7-9. What is the main material of the exterior walls of your household?

NATURAL WALLS 11) No walls 12) Cane / Palm / Trunks 13) Dirt / Landcrete

RUDIMENTARY WALLS 21) bamboo with mud 22) Stone with mud 23) Uncovered adobe

24) Plywood 25) Cardboard 26) Reused wood

FINISHED WALLS 31) Cement 32) Stone with lime / Cement 33) Bricks 34) Cement blocks 35) Covered adobe 36) Wood planks / Shingles

96) OTHER (Specify ____________________ )

7-10. Does any member of this household own any agricultural land?

1) Yes 2) No

7-11. Does your household have

| **a) Electricity?** | **1) Yes 2) No** |
| --- | --- |
| **b) A wall clock** | 1) Yes 2) No |
| **c) A radio?** | 1) Yes 2) No |
| **d) A black/white television?** | 1) Yes 2) No |
| **e) A color television?** | 1) Yes 2) No |
| **f) A mobile telephone?** | 1) Yes 2) No |
| **g) A land-line telephone?** | 1) Yes 2) No |
| **h) A refrigerator?** | 1) Yes 2) No |
| **i) A freezer?** | 1) Yes 2) No |
| **j） Electric generator/Inventor(s)？** | 1) Yes 2) No |
| **K） Washing machine？** | 1) Yes 2) No |
| **l) Computer/Tablet computer?** | 1) Yes 2) No |
| **m) Photo camera? (NOT ON PHONE)?** | 1) Yes 2) No |
| **n) Video deck/DVD/VCD?** | 1) Yes 2) No |
| **o) Sewing machine?** | 1) Yes 2) No |
| **p) Bed?** | 1) Yes 2) No |
| **q) Table?** | 1) Yes 2) No |
| **r) Cabinet/Cupboard?** | 1) Yes 2) No |
| **s) Access to the Internet in any device?** | 1) Yes 2) No |

7-12. Does any member of this household own

| **a) A wrist watch?** | **1) Yes 2) No** |
| --- | --- |
| **b) A bicycle?** | 1) Yes 2) No |
| **c) A motorcycle or motor scooter?** | 1) Yes 2) No |
| **d) An animal-drawn cart?** | 1) Yes 2) No |
| **e) A car or truck?** | 1) Yes 2) No |
| **f) A boat with a motor?** | 1) Yes 2) No |
| **g) A boat without a motor?** | 1) Yes 2) No |

**Thank you very much for your cooperation.**
